# Testing the effects of combining azithromycin with inhaled tobramycin for *P. aeruginosa* in cystic fibrosis; a randomized, controlled clinical trial

**DOI:** 10.1136/thoraxjnl-2021-217782

**Published:** 2021-10-27

**Authors:** David P Nichols, Pradeep K Singh, Arthur Baines, Lindsay J Caverly, James F Chmiel, Ronald L Gibson, Jorge Lascano, Sarah J Morgan, George Retsch-Bogart, Lisa Saiman, Hossein Sadeghi, Joanne L Billings, Sonya L Heltshe, Shannon Kirby, Ada Kong, Jerry A Nick, Nicole Mayer-Hamblett, TEACH Study Group

**Affiliations:** 1 Department of Pediatrics, University of Washington School of Medicine. Seattle, WA USA; 2 Seattle Children’s Research Institute, Seattle, WA USA; 3 Department of Microbiology, University of Washington. Seattle, WA USA; 4 Department of Pediatrics, University of Michigan Medical School. Ann Arbor, MI USA; 5 Department of Pediatrics, Indiana University School of Medicine. Indianapolis, IN. USA; 6 Department of Medicine, University of Florida Health. Gainesville, FL USA.; 7 Department of Pediatrics, University of North Carolina at Chapel Hill School of Medicine. Chapel Hill, NC USA; 8 Department of Pediatrics, Columbia University Medical Center. New York, NY USA; 9 Department of Medicine, University of Minnesota Medical Center. Minneapolis, MN USA; 10 Department of Biostatistics, University of Washington School of Public Health, Seattle, Washington, USA; 11 Department of Pharmacy, Seattle Children’s Hospital, Seattle, Washington, USA; 12 Department of Medicine, University of Colorado School of Medicine, Aurora, Colorado, USA; 13 Department of Medicine, National Jewish Health, Denver, Colorado, USA

**Keywords:** cystic fibrosis, Pseudomonas, tobramycin, azithromycin, drug-drug interaction

## Abstract

**Rationale:**

Inhaled tobramycin and oral azithromycin are common chronic therapies in people with cystic fibrosis and *P. aeruginosa* airway infection. Some studies have shown that azithromycin can reduce the ability of tobramycin to kill *P. aeruginosa*. This trial was done to test the effects of combining azithromycin with inhaled tobramycin on clinical and microbiological outcomes in people already using inhaled tobramycin. We theorized that those randomized to placebo (no azithromycin) would have greater improvement in FEV_1_ and greater reduction in sputum *P. aeruginosa* in response to tobramycin.

**Methods:**

6-week prospective, randomized, placebo-controlled, double-blind trial testing oral azithromycin vs. placebo combined with clinically prescribed inhaled tobramycin in individuals with cystic fibrosis and *P. aeruginosa* airway infection.

**Results:**

Over a 6-week period including four weeks of inhaled tobramycin, the relative change in FEV_1_ did not statistically significantly differ between groups (azithromycin (n=56) minus placebo (n=52) difference 3.44%; 95% CI: −0.48, 7.35; p=0.085). Differences in secondary clinical outcomes, including patient reported symptom scores, weight, and need for additional antibiotics, did not significantly differ. Among the 29 azithromycin and 35 placebo participants providing paired sputum samples, the 6-week change in *P. aeruginosa* density differed in favor of the placebo group (difference 0.75 log_10_ CFUs/mL; 95% CI: 0.03, 1.47; p=0.043).

**Conclusions:**

Despite having greater reduction in *P. aeruginosa* density in participants able to provide sputum samples, participants randomized to placebo with inhaled tobramycin did not experience significantly greater improvements in lung function or other clinical outcomes compared to those randomized to azithromycin with tobramycin.

## Background

Two of the earliest pulmonary drug therapies proven effective for people with cystic fibrosis (PwCF) are inhaled tobramycin and oral azithromycin^1 2^. The CF National Patient Registry in the US reports that these two antibiotics are used in approximately two-thirds of PwCF who have persistent *P. aeruginosa* (*Pa*) airway infection, and the majority use them in combination^3^. However, our prior *in vitro* and murine model studies found that azithromycin potently reduced the antibacterial effect of tobramycin against *Pa* ^4–6^. Moreover, *post-hoc* analyses of clinical and research databases indicated that individuals on chronic oral azithromycin may benefit less from inhaled or intravenous tobramycin when compared to those not using this macrolide therapy^4–10^. Given the widespread concomitant use of both drugs and desire to not rely on *in vitro* or *post-hoc* analyses, a prospective, randomized, placebo-controlled clinical trial was conducted to test if the absence of concomitant azithromycin improved the clinical benefits and *Pa* killing expected from inhaled tobramycin (NCT02677701). The primary hypothesis tested in this trial was that those randomized to receive placebo (vs. azithromycin) with inhaled tobramycin would experience greater increase in lung function measured by forced expiratory volume in one second (FEV_1_). Secondarily we tested the impact on other clinical outcomes and the hypothesis that those given placebo would experience greater reduction in *Pa* sputum density. Some of the results have been presented as an abstract^11^.

## Methods

### Study Design

**Figure 1:**
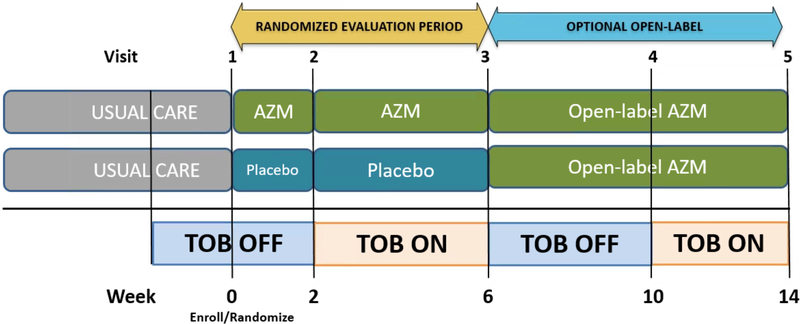
Study design schematic. The study consisted of three visits during the 6-week randomized period. Participants were randomly allocated 1:1 to receive oral azithromycin (500 mg) or placebo three times per week throughout the 6-week period. Two weeks post-randomization, participants started their usual inhaled tobramycin solution or powder twice daily for 4 weeks while continuing on study drug (azithromycin or placebo, see on-line suppl for details) to complete the 6-week study. Participants completing the study were offered optional, additional participation in an 8-week open-label period.

The TEACH study was a prospective, randomized, placebo-controlled, double-blinded, clinical trial investigating the effects of oral azithromycin in combination with inhaled tobramycin on clinical and microbiologic outcomes among PwCF and *Pa* airway infection. Figure 1 displays the overall study design for the 6-week randomized study followed by an optional open label period, the results of which will be published separately. Participants were randomized 1:1 to azithromycin 500mg thrice weekly or placebo at week 0, which was followed two weeks later by initiation of their prescribed inhaled tobramycin for an additional four weeks. An adaptive algorithm was used to balance randomization by FEV_1_ % predicted (25%−50%, >50%−75%, >75%), chronic oral azithromycin use for the past 30 days (yes/no), inhaled tobramycin formulation (TIS or TIP), and site^12–14^.

Participants were ≥12 years old with CF and otherwise clinically stable with: ≥2 *Pa* positive respiratory cultures in the last year (one of which was within the last 6 months), percent of predicted FEV_1_ (ppFEV_1_) 25–100%, and current or prior chronic use of oral azithromycin (detailed criteria; Table E1). Participants must have used ≥2 cycles of inhaled tobramycin (4 weeks per cycle) in the 6 months prior to enrollment. The trial (NCT02677701) was conducted at 39 CF Foundation accredited Care Centers in the United States, was approved by central or local institutional review boards, was coordinated by the CF Therapeutics Development Network Coordinating Center (TDNCC; Seattle, WA), and was monitored by a Data Safety Monitoring Board (DSMB) appointed by the National Heart, Lung, and Blood Institute.

Pulmonary function testing, anthropometric measures, and patient-reported respiratory symptoms were collected at all study visits. Expectorated sputum collection was attempted at all study visits for quantitative *Pa* culture. Adherence to inhaled tobramycin and study drug were collected using participant daily diaries. Adherence to study drug was also assessed using the number of study drug capsules remaining in study drug bottles.

### Study Endpoints

The primary endpoint was the relative change in FEV_1_ liters, from baseline (week 0) to week 6, which included the necessary two-week period post-randomization important for either wash-out or wash-in of azithromycin prior to the initiation of inhaled tobramycin. A key secondary endpoint was the relative change in FEV_1_ liters from week 2 to week 6 when participants were taking inhaled tobramycin in addition to study drug (azithromycin vs. placebo).

Additional secondary clinical endpoints included: changes in ppFEV_1_ (Global Lung Initiative reference equations^15^); changes in weight; need for acute intravenous, oral, or inhaled antibiotics or hospitalization during the study; and incidence of pulmonary exacerbation^16 17^. Patient-reported secondary endpoints were changes in the Cystic Fibrosis Questionnaire – Revised: Respiratory Symptom Score (CFQ-R RSS) and the Cystic Fibrosis Respiratory Symptom Diary – Chronic Respiratory Infection Symptom Score (CFRSD-CRISS)^18 19^

The key microbiological endpoint, an additional secondary endpoint, was change in *Pa* sputum density from baseline to week 6. The change from week 2 to week 6 (inhaled tobramycin use) was also determined. Sputum samples were immediately processed and frozen at study sites before being shipped to a blinded, centralized microbiology laboratory (see Online Supplement). Safety endpoints included rates of adverse events including QTc > 500 milliseconds or increase in QTc of ≥ 60 milliseconds.

### Statistical Analysis

Analyses were performed on the modified intent-to-treat (m-ITT) population, defined as all randomized participants who received ≥1 dose of study drug. Analysis of the primary endpoint was repeated using the per-protocol (PP) population, defined as participants in the m-ITT population who completed ≥80% of their doses of study drug, did not require use of acute antibiotics or steroids, and had no major protocol violations. Analysis of the microbiology endpoint was performed on the subset of the m-ITT population from whom paired expectorated sputum samples were collected to measure change in *Pa* density.

The primary endpoint was compared between treatment groups using a linear regression model adjusted for ppFEV_1_ (25%−50%, >50%−75%, >75%), azithromycin use at baseline, and tobramycin formulation (inhaled tobramycin formulation: powder [TIP] vs. solution [TIS]). Continuous secondary endpoints were modeled similarly to the primary endpoint. Counts and percentages were summarized and Fisher’s exact tests with corresponding 95% confidence intervals (CIs) derived from the Newcombe-Wilson method without continuity correction were used to compare treatment groups. Rate ratios were estimated using Poisson regression with an offset of the logarithm of observation time.

A two-sided, 0.05 significance level was used. With 120 participants assuming 10% attrition, the study had 85% power to detect a treatment effect of 7.5% or greater in the relative change in FEV_1_ liters using an estimated standard deviation (SD) of 13 for FEV_1_^1 2^. There was no alpha adjustment for multiple testing for secondary efficacy variables. P-values from these tests were considered descriptive and evaluated for nominal significance only when p<0.05. Interim monitoring for efficacy/harm was performed by the DSMB at pre-specified time points after 50% and 75% of participant completion.

## Results

### Study Population

**Figure 2:**
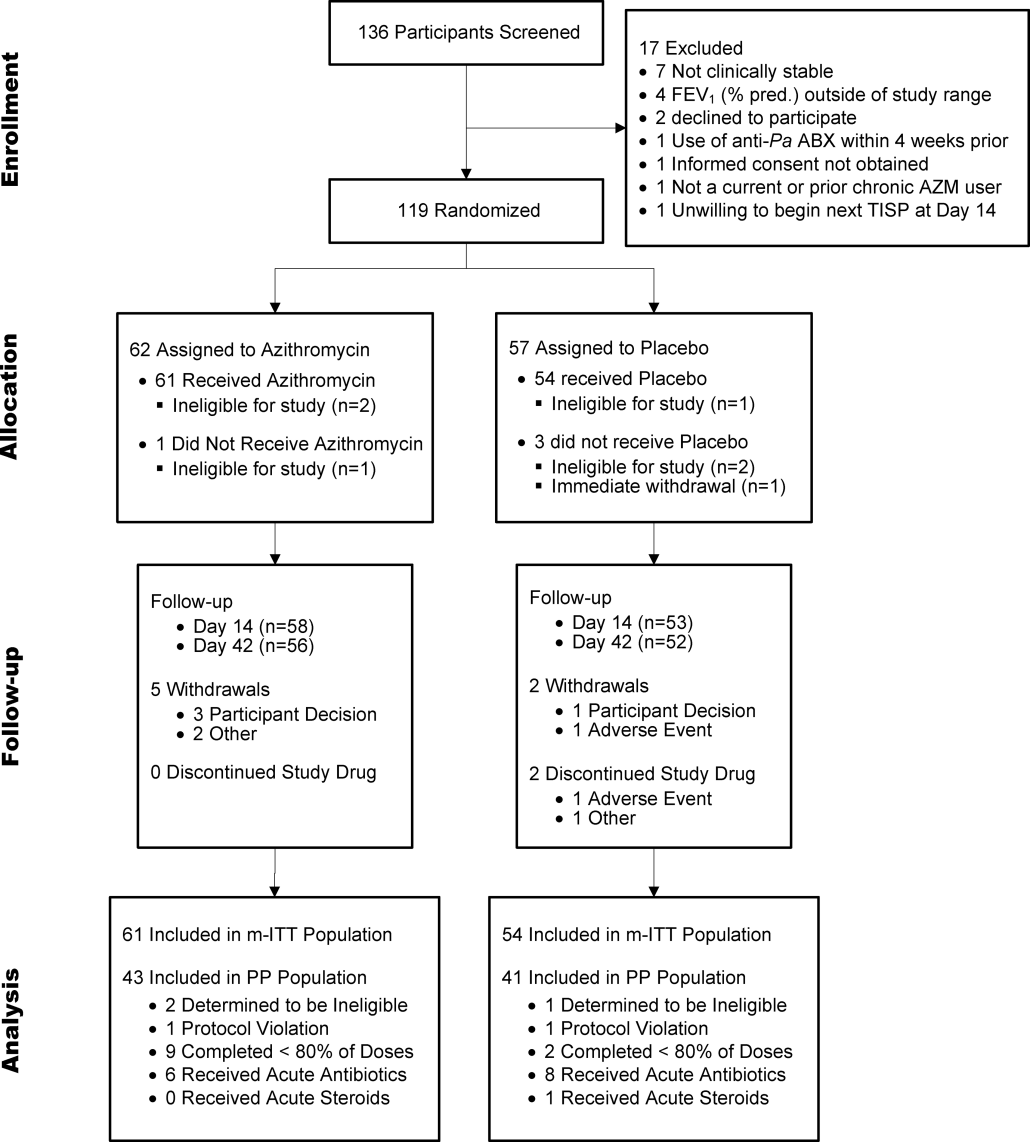
Overview of the study population. Individuals screened, randomized, length of follow up, and included in the analytical populations.

Between October 2016 and December 2019, 136 participants screened for study eligibility and 119 participants were randomized: 57 to placebo and 62 to azithromycin. Four participants did not receive study drug and were not replaced. Three were determined ineligible before their first dose and one voluntarily withdrew (Figure 2).

Demographics and baseline characteristics for the 115 randomized and treated participants (ITT population) were similar between groups (Table 1). The azithromycin group had slightly more heterozygous for *F508del* (28%) than the placebo group (20%). Mean FEV_1_ was 2.59 liters (SD=0.81) in the azithromycin group and 2.50 (SD=0.85) in the placebo group. Chronic medication use was comparable, though more placebo participants used CFTR modulators (69% versus 51% in the azithromycin group).

Of the 115 randomized and treated participants, five from the azithromycin group and two from the placebo group withdrew from the study early. Two additional placebo participants discontinued study drug permanently while enrolled (Figure 2). Mean follow-up time was similar, averaging 6.1 weeks in the azithromycin group and 6.4 weeks in the placebo group.

Adherence to three times weekly study drug self-administration (azithromycin or placebo) was 90.9% (SD=21.3) of expected doses in the azithromycin group and 95.8% (SD=15.3) in the placebo group. Average adherence to twice daily inhaled tobramycin solution was 85.3% (SD=21.7) in the azithromycin group and 87.8% (SD=17.7) in the placebo group. Adherence for participants using tobramycin inhalation powder was 85.5% (SD=16.5) in the azithromycin group and 92.1% (SD=11.8) in the placebo group.

### Pulmonary Function

**Figure 3:**
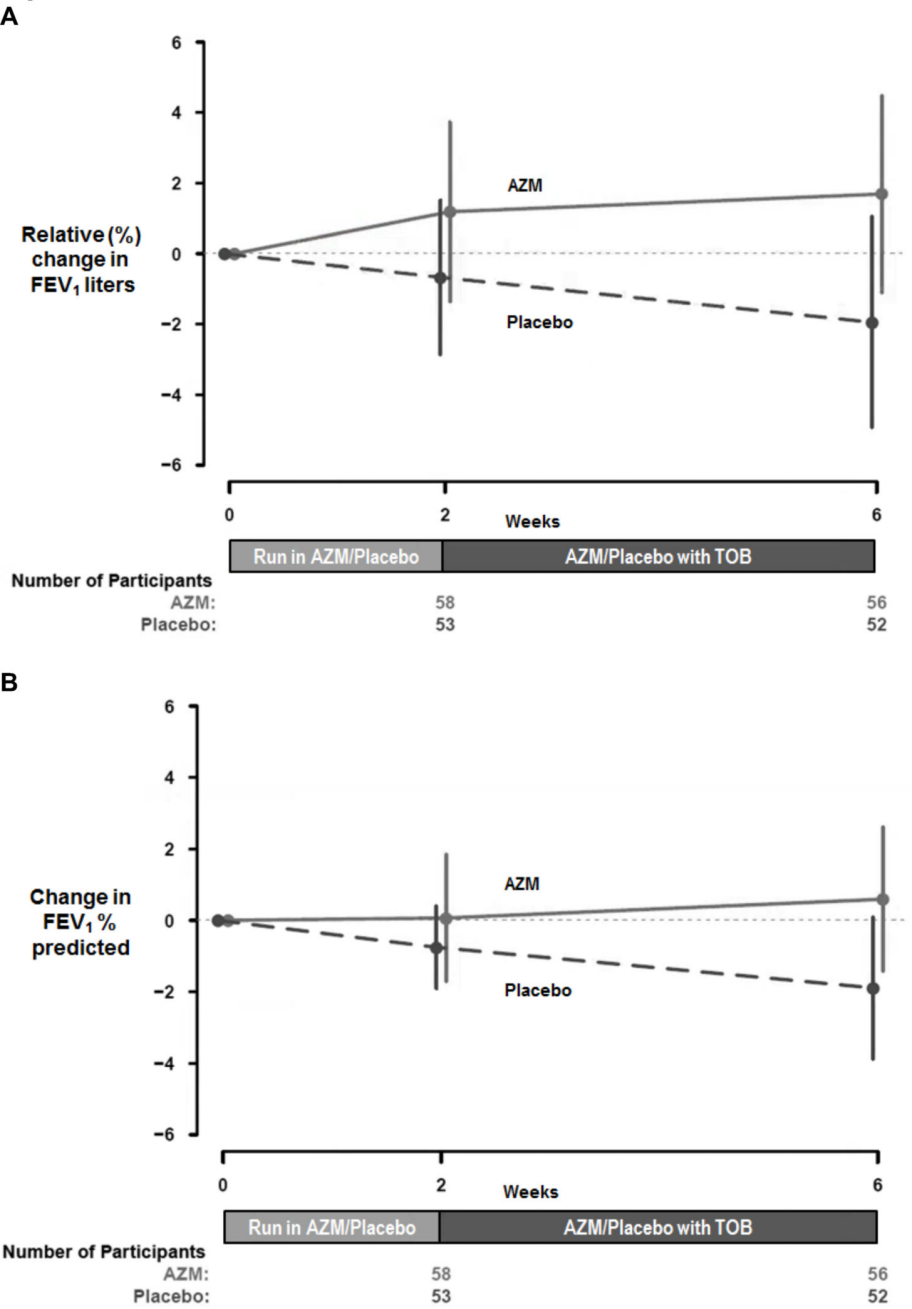
Pulmonary function outcomes: (A) Mean relative (%) change from baseline in FEV_1_ liters, and (B) Mean absolute change from baseline in ppFEV1. Error bars are 95% confidence intervals, AZM=Azithromycin.

**Figure 4:**
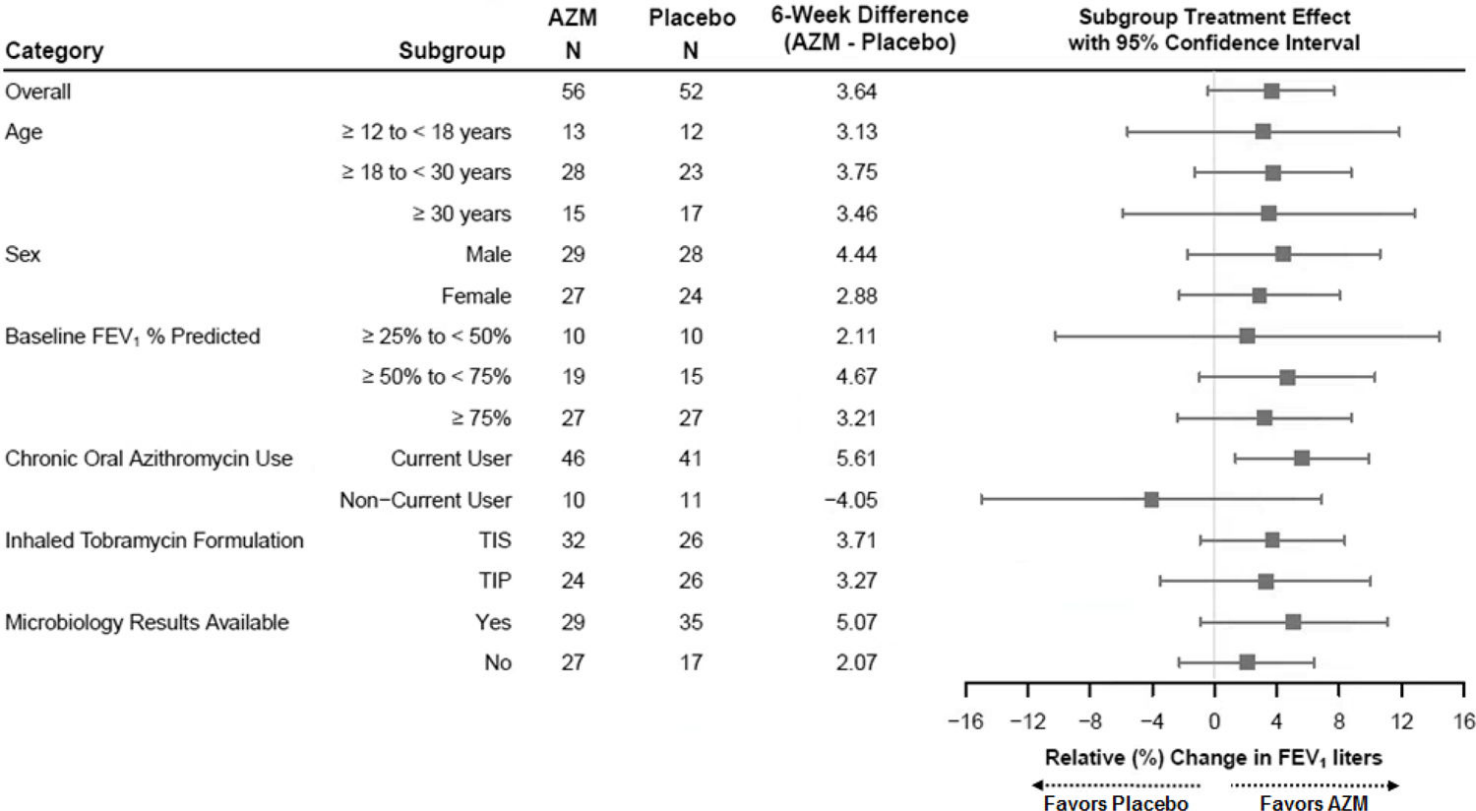
Mean relative (%) change from baseline in FEV_1_ liters (unadjusted estimates) among pre-specified subgroups, AZM=Azithromycin.

108 study participants completed spirometry at both baseline and Week 6 end-of-study visits, comprising the primary m-ITT population (56 azithromycin, 52 placebo). There was an average 1.69% (SD=10.39) relative change in FEV_1_ liters at Week 6 in the azithromycin group and an average −1.95% (SD=10.73) relative change in FEV_1_ liters in the placebo group (Figure 3A), which did not significantly differ between groups (mean difference adjusted for stratification factors 3.44%; 95% CI: −0.48, 7.35; p=0.085). Analysis of the primary endpoint in the per-protocol study population was similar with a mean difference of 3.54% (95% CI: −0.82, 7.91; p=0.110). Additional sensitivity analyses of the primary endpoint and individual participant data are included in Figures E1 and E2. Pre-specified unadjusted subgroup analyses of the primary outcome (Figure 4) were generally consistent with that of the overall study cohort across subgroups.

During the inhaled tobramycin period of the study (Week 2 to Week 6), the difference between treatment groups in mean relative change in FEV_1_ liters was not significant. The estimated treatment difference, adjusted for lung function at Week 2, was 1.36% (95% CI: −2.55, 5.27; p=0.491). The mean 6-week absolute change in ppFEV_1_ was 0.6% (SD=7.5) and −1.9% (SD=7.1) in the azithromycin and placebo groups, respectively (Figure 3B, mean difference adjusted for randomization strata of 2.28%; 95% CI: −0.42, 4.98; p=0.097). During the inhaled tobramycin portion of the study the estimated treatment difference in ppFEV_1_, adjusted for lung function at Week 2, was 1.45% (95% CI: −0.94, 3.84; p=0.232).

### Secondary Clinical Outcomes

Changes in weight (kg) were similar between the two treatment groups, with a mean change from baseline to Week 6 of 0.23 kg (SD=1.44) in the azithromycin group and −0.02 kg (SD=1.40) in the placebo group (Figure E3, mean difference adjusted for randomization strata 0.20 kg, 95% CI: −0.33, 0.74; p= 0.454). The proportions of participants with any antibiotic use (oral, inhaled, or intravenous route), any pulmonary exacerbations, and any hospitalizations during the study were comparable between the two groups (Table 2).

Neither of the two patient-reported outcomes measuring respiratory symptoms showed significant differences in mean scores from baseline to Week 6 (Figure E4). The CFQ-R RSS (higher score indicates fewer symptoms) had an adjusted treatment difference of 1.53 points (95% CI: −3.70, 6.77; p=0.563), comparing azithromycin to placebo. While the CFRSD-CRISS (lower score indicates fewer symptoms) had an adjusted treatment difference of −2.89 points (95% CI: −7.01, 1.22; p=0.166). In both measures, the scores trended in favor of the azithromycin group.

### Secondary Microbiologic Outcomes

The ability to produce sputum samples and rates of culture positivity for *Pa* across timepoints varied among participants. Over 80% of sputum samples grew *Pa* at baseline, and this was similar between the two groups. Overall, 29 of 61 (47.5%) azithromycin and 35 of 54 (64.8%) placebo participants were able to produce expectorated sputum sufficient for culture at both baseline and Week 6 study visits, corresponding to 56% of the total ITT population. This subgroup was, on average, a year older and had 5.6% lower baseline ppFEV_1_ than the ITT population. The azithromycin and placebo groups providing these microbiological data had similar baseline characteristics (Table E2). For comparison purposes, the difference in the 6-week relative change in FEV_1_ among this subgroup (azithromycin minus placebo, unadjusted) was 5.07% (95% CI: −0.95, 11.08) as compared to those unable to produce sputum (27 azithromycin; 17 placebo) who experienced a 2.07% difference in the relative change in FEV_1_ (95% CI: −2.26, 6.40). (Figure 4). In those able to provide sputum samples for culture, the differences in change in FEV_1_ developed almost entirely between Weeks 0–2 before starting inhaled tobramycin (Figure E5).

The azithromycin group had an average 6-week change from baseline of +0.30 log_10_ (CFUs/mL) (SD=1.69) in *Pa* sputum density and the placebo group had an average change of −0.49 log_10_ (CFUs/mL) (SD=1.20). The mean difference between groups in the 6-week change, adjusted for stratification factors, was 0.75 log_10_ (CFUs/mL) (95% CI: 0.03, 1.47; p=0.043; Figure 3). During the 4-week inhaled tobramycin period, the mean difference in change in log_10_ (CFUs/mL) *Pa* density was 0.64, 95% CI: −0.01, 1.28; p=0.053). The proportion of participants *Pa* positive at each study visit and relative changes in lung function (FEV_1_ liters) among the subgroup providing sputum samples are further described in supplemental Table E3 and Figures E5 and E6, respectively.

Over 80% of participants reported using chronic azithromycin for ≥30 days at the time of enrollment. This large subpopulation represented common clinical care practice in many countries. Exploratory subgroup analyses were performed to characterize the differences in changes in FEV_1_ and *Pa* density between treatment groups when considering chronic azithromycin use prior to enrollment. (Table E4, unadjusted data). Those entering the trial using azithromycin and able to produce sputum samples had a difference in the 6-week relative change in FEV_1_ of 7.18% (95% CI: 1.05, 13.32) favoring azithromycin but no difference in the 4-week change during inhaled tobramycin (−0.13%; 95% CI −6.76, 6.50). Among these participants, the difference in the 6-week change in *Pa* density was 1.01 log_10_ (CFUs/mL) (95% CI: 0.18, 1.85) favoring placebo and the 4-week change during inhaled tobramycin favoring placebo was 0.71 log_10_ CFUs/mL (95% CI: −0.11, 1.53). See Table E4 for additional details, including data from the small group not using azithromycin at enrollment.

### Safety

Rates of serious adverse events (SAEs) did not significantly differ between the two treatment groups, 4 participants in the azithromycin group experienced 10 SAEs during the study, while 3 participants in the placebo group experienced 14 SAEs (rate ratio adjusted for follow-up time: 0.67; 95% CI: 0.29, 1.50).

Fewer total adverse events (AEs) were observed in the azithromycin group during the study, with 109 AEs among 40 participants in the azithromycin group and 136 AEs among 38 participants in the placebo group (rate ratio adjusted for follow-up time: 0.75; 95% CI: 0.58, 0.97; p=0.026). A large percentage of AEs were attributed to respiratory, thoracic and mediastinal disorders with 22 participants in the azithromycin group experiencing 42 AEs and 30 participants in the placebo group experiencing 74 AEs (Table E5, rate ratio adjusted for follow-up of 0.53; 95% CI: 0.36, 0.77). No participants in either treatment group were found to have abnormal QTc intervals measured via electrocardiograms during the study.

## Discussion

The TEACH trial was designed to test the impact of using concomitant azithromycin on the clinical response to ongoing inhaled tobramycin, in addition to its impact on sputum *Pa* density over a 6-week period. The rationale for this trial emerged from *in vitro* studies and several *post-hoc* clinical data analyses suggesting that PwCF using chronic azithromycin may respond less favorably to inhaled tobramycin compared with those not using azithromycin^4 5 7–10^. In this prospective trial, we hypothesized that the placebo group would be superior to the azithromycin group when testing clinical and microbiological outcomes across a 6-week period that included a 4-week cycle of inhaled tobramycin therapy. We found that placebo-treated participants did not experience greater improvement in FEV_1_ or other clinical outcomes. This was despite the fact that placebo-treated participants able to provide sputum samples had greater reduction in *Pa* sputum density (i.e., bacterial killing) compared with those randomized to azithromycin.

TEACH is one of few prospective, randomized, placebo-controlled trials to examine the potential for an adverse interaction between proven and widely used therapies in CF. Strengths of the study include the clinical relevance of the tested hypothesis, prospective multi-center conduct with randomization, blinding with a placebo comparison, and a representative participant population. It seems increasingly important to consider whether individual or combined chronic therapies may be less useful than anticipated as the CF community benefits from better overall health and prioritizes such research^20 21^. This not only helps to reduce daily treatment burden by working to identify those therapies that remain effective in long-term use, but also opens space to develop new and more effective drugs.

In TEACH, the 6-week change in lung function (FEV_1_) was the primary test for clinical benefit and primary outcome of the trial (Figure 1). There was a trend toward better FEV_1_ in the azithromycin arm compared with placebo. No significant differences occurred among secondary clinical outcomes, including measures of patient reported respiratory symptom scores, weight, or need for additional antibiotics. The azithromycin group experienced statistically fewer adverse events, though what this means in the absence of differences in pulmonary exacerbations or antibiotic use is unclear.

Study outcomes focused on the change over the entire 6 weeks in order to maintain baseline similarities between groups achieved at randomization (week 0) while enabling wash-in or washout of azithromycin for 2 weeks prior to starting inhaled tobramycin. Based on pharmacokinetics data, 2 weeks was identified as sufficiently long to reach very low concentrations of azithromycin in the airway^22–25^. Some studies find that azithromycin can be measured within leukocytes in the lung beyond 14 days, but the combined clinical and microbiological results from this trial suggest that two weeks was adequate to test for the hypothesized interaction with tobramycin as observed *in vitro*.

At enrollment, 80% of participants reported using chronic azithromycin and so relatively few were started on macrolides while most of those randomized to placebo had macrolide therapy functionally withdrawn. Azithromycin by itself has been shown to improve FEV_1_ in CF lung disease, especially those with *Pa* infection^2 26 27^. Thus, the small FEV_1_ changes during the trial, mostly seen as a decline in FEV_1_ in the placebo group, may primarily represent the impact of azithromycin. This interpretation is supported by the fact that much of the change in FEV_1_ from baseline occurred over the first two weeks after randomization, which was prior to starting inhaled tobramycin (Figure 3, Table E4).

**Figure 5:**
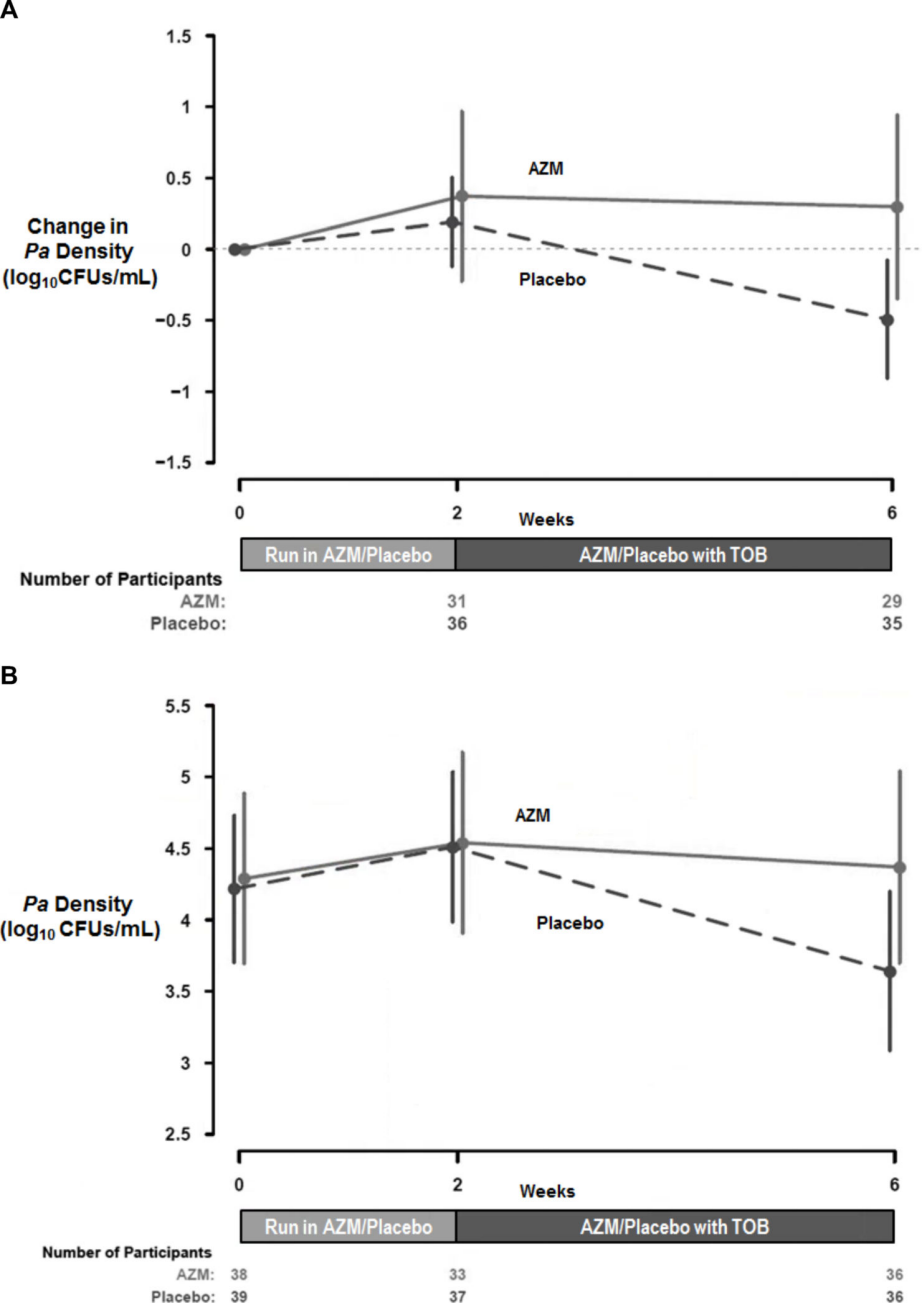
Microbiologic outcomes: (A) Mean change from baseline in *Pa* density in log_10_(CFUs/mL) and (B) Mean *Pa* density in log_10_(CFUs/mL). Error bars are 95% confidence intervals, AZM=Azithromycin.

As a secondary aim, the TEACH trial tested whether those randomized to placebo vs. azithromycin would experience greater reduction in sputum *Pa*, which would be consistent with *in vitro* antagonism between these two antibiotics^4–6^. As theorized, those randomized to placebo had a greater decrease in sputum *Pa* density, suggesting greater ability of inhaled tobramycin to kill *Pa* in the CF airway if azithromycin is not present (Figures 5, E6). This difference occurred mostly during inhaled tobramycin period (week 2–6). The treatment effect size of 0.75 log_10_ CFU/mL of sputum is similar in magnitude to the effect of inhaled tobramycin on sputum *Pa* density in other trials enrolling participants already using inhaled tobramycin^28–31^. These microbiological data should be placed in context with the lack of greater clinical benefits (e.g. lung function, respiratory symptoms) with placebo and the reduced number of participants able to produce sputum samples for quantitative culture. Modest differences in baseline characteristics and other outcomes measured between those able or unable to provide sputum are provided in the on-line supplement (Tables E2, E4).

Additional potential limitation when interpreting study results include the relatively small differences in 6-week and 4-week changes in FEV_1_ in both groups, which were variable (Figure E2) and not statistically significantly different between groups. Changes in lung function should not be overinterpreted beyond the lack of superiority with placebo, a finding that was counter to our hypothesis. However, the 95% confidence interval for the primary outcome of relative change in FEV_1_ favoring azithromycin (−0.44% to 7.35%) makes it highly unlikely that using azithromycin results in lesser improvement in FEV_1_ during continued cycles of inhaled tobramycin. This trial was also not designed or powered for subgroup analyses (e.g. prior use of azithromycin), and those results should be viewed as exploratory. Lastly, we enrolled people already using inhaled tobramycin therapy and the study was not designed to determine any impact of azithromycin on the initial response to inhaled tobramycin or long-term effects of chronic therapy (e.g. risk of acute pulmonary exacerbation or rate of decline in lung function).

Studies of inhaled antibiotics in CF have reported poor correlation between improved FEV_1_ and reduced *Pa* sputum density when considering individual participants (Figure E7)^32 33^, but at the level of treatment groups (e.g. inhaled antibiotics vs. placebo), most studies in PwCF find greater increase in FEV_1_ in the group with greater reduction in sputum bacterial density^1 34^. This did not occur in our trial, similar to what has been seen in studies of inhaled levofloxacin in CF and multiple inhaled antibiotics in non-CF bronchiectasis^30 35^. One potential explanation is that beneficial effects of azithromycin in the airway unrelated to pseudomonas outweighed the effects of increased *Pa* burden, resulting in a disconnect between changes in lung function and airway infection^36–40^. Another potential explanation is that our study population, by design, was not naïve to inhaled tobramycin, and neither group (i.e., azithromycin or placebo-treated) had a significant increase in FEV_1_ during inhaled tobramycin use. A diminishing effect on FEV_1_ over subsequent cycles of inhaled tobramycin was reported in even the earliest clinical trials^1^. It would be interesting to conduct this trial in a population with *Pa* without prior exposure to inhaled tobramycin and in which larger impacts on FEV_1_ may be expected. This was not feasible in the US but may be possible in other regions where tobramycin use is less common. Our trial, conducted in a population with substantial prior drug exposure, serves to highlight uncertainty about the sustained clinical effect of certain chronic CF medications and how best to measure this. Future research may need to more directly quantify the health benefits afforded by common daily therapies as more PwCF express interest in reducing burden of care^20 21^. More specifically, the disconnection between clinical and microbiological outcomes in this trial suggests that better understanding of chronic antimicrobial therapies is needed, in light of treatment burden, cost, potential toxicity, and antibiotic stewardship.

Other researchers have reported changes in sputum microbiome during inhaled antibiotics, suggesting that microbiological effects on species other than *Pa* may also be important in the clinical response^41 42^. This is interesting to consider as an alternative explanation for the lack of association between change in *Pa* and lung function or other clinical outcomes; however, the investigators reporting microbiome changes similarly found no mean improvement in lung function after 4 weeks of inhaled tobramycin, indicating that the clinical implications of changing sputum microbial ecology through inhaled antibiotics requires further study^41^.

Ultimately, our trial clearly demonstrated that eliminating concomitant azithromycin does not result in greater clinical response to ongoing, chronic inhaled tobramycin over the short term, despite changes in sputum *Pa* density that are consistent with antagonism between these two antibiotics in the CF airway. Additional outcomes such as rate of FEV_1_ decline, risk of acute pulmonary exacerbations, and survival are of great interest but may be increasingly difficult to include in a prospective, randomized study as increasing numbers of PwCF are fortunately experiencing more stable pulmonary health and fewer exacerbations than in the past^43 44^. Several years ago, inhaled tobramycin use in the US CF patient registry was shown to associate with improved survival^45^. More recently, analyses of CF patient registries in the US and France found that inhaled tobramycin and azithromycin each associated with slower rate of decline in FEV_1_; however, some of these data also suggest that both medications combined may be less effective^7 27 46^. The TEACH trial, while reassuring regarding any short-term clinical effects of combined drug use, was not designed to determine such long-term outcomes.

This study represents the first multicenter trial in those with CF chronically infected with *Pa* to directly assess clinical and microbiological outcomes associated with the combined use of azithromycin and inhaled tobramycin. While benefit from broad-spectrum antibiotics can extend far beyond antibacterial effects against a specific pathogen, they must be balanced with off-target effects that are complex and often difficult to identify. Like many trials, our findings raise additional questions, including the long-term clinical relevance of microbiological changes in the airway that occur with chronic antibiotic use. As the landscape of CF treatment and care evolves, in particular the expanding use of CFTR modulators, trials similar to TEACH will be necessary to determine the optimal and least burdensome treatment approaches.

## Supplementary Material

Supp1

## Figures and Tables

**Table 1.** Participant baseline characteristics and demographics by treatment group.

Characteristic	Azithromycin (N = 61)	Placebo (N = 54)
Age, years	26.1±9.9	265±9.7
Age, no. (%)		
≥ 12 to < 18 years	14 (23.0)	12 (22.2)
≥ 18 to < 30 years	28 (45.9)	25 (46.3)
≥ 30 years	19 (31.1)	17 (31.5)
Female, no. (%)	29 (47.5)	26 (48.1)
Race, no. (%)		
Caucasian	55 (90.2)	49 (90.7)
Other*	6 (98)	5 (9.3)
Ethnicity, no. (%)		
Hispanic or Latino	9 (14.8)	7 (13.0)
FEV_1_, liters	2.59±0.81	2.50±0.85
FEV_1_, % predicted †	70.7±18.2	69.6±21.1
FEV_1_, % predicted category, n (%)†		
≥ 25% to < 50%	11 (18.0)	11 (20.4%)
≥ 50% to < 75%	22 (36.1)	16 (29.6%)
≥ 75%	28 (45.9)	27 (50.0%)
Height, cm	167.9±10.2	166.4±9.6
Weight, kg	63.9±13.8	62.7±13.2
Genotype, no. (%)		
F508del homozygous	38 (62.3)	35 (64.8)
F508del heterozygous	17 (27.9)	11 (20.4)
Other	6 (9.8)	7 (13.0)
Unavailable	0 (0)	1 (1.9)
Tobramycin Formulation, no. (%)		
Solution	33 (54.1)	28 (51.9)
Powder	28 (45.9)	26 (48.1)
History of Azithromycin Use, no. (%)		
Current Chronic User	51 (83.6)	43 (79.6)
Non-Current Chronic User	10 (16.4)	11 (20.4)
Chronic Medication Use, no. (%)		
Dornase Alfa	53 (86.9)	48 (88.9)
Hypertonic Saline	46 (75.4)	40 (74.1)
High Dose Ibuprofen	2 (3.3)	2 (3.7)
Ivacaftor	2 (3.3)	3 (5.6)
Ivacaftor/Lumacaftor	14 (23.0)	18 (33.3)
Ivacaftor/Tezacaftor	14 (23.0)	16 (29.6)
Elexacaftor/Tezacaftor/Ivacaftor	1 (1.6)	0 (0)
*Pa* Sputum Density		
Participants with Sputum Culture Results, no. (%)	38 (62.3)	39 (72.2)
*Pa* log_10_(CFU/mL)	4.29±1.80	4.22±1.59

Plus-minus values are mean ± SD.

* Other includes Black/African American, American Indian/Alaska Native, Asian, Native Hawaiian/Pacific Islander, Unknown, and Other.

† Percent predicted calculated using Global Lung Initiative reference equations.

**Table 2.** Antibiotic use, pulmonary exacerbations, and hospitalizations by treatment group.

	Azithromycin (N = 61)	Placebo (N = 54)	Difference (95% CI)

Oral Antibiotic Use			
Participants with ≥1 event, no. (%)	7 (11.5%)	11 (20.4%)	−8.9% (−22.7%, 4.6%)

Intravenous Antibiotic Use			
Participants with ≥1 event, no. (%)	3 (4.9%)	2 (3.7%)	1.2% (−8.2%, 10.2%)

Inhaled Antibiotic Use (other than tobramycin)			
Participants with ≥1 event, no. (%)	1 (1.6%)	2 (3.7%)	−2.1% (−11.0%, 5.5%)

Pulmonary Exacerbation			
Participants with ≥1 event, no. (%)	9 (14.8%)	8 (14.8%)	−0.1% ( −13.7%, 13.0%)

Hospitalization			
Participants with ≥1 event, no. (%)	3 (4.9%)	3 (5.6%)	−0.6% (−10.7%, 8.7%)

## Key Messages:

### What is the key question:

This trial tested whether or not using azithromycin diminished the short-term clinical or microbiological effects of ongoing inhaled tobramycin in people with cystic fibrosis.

### What is the bottom line:

Despite a greater reduction in sputum density of *P. aeruginosa*, people treated with placebo compared with azithromycin did not have greater improvement in lung function or disease-related quality of life during treatment with inhaled tobramycin.

### Why read on:

This is one few trials to consider the impact of approved drugs in combination in CF and provides some reassurance for a common clinical practice pattern while identifying the need to better understand long-term effects of chronic therapies and the clinical relevance of microbiological changes in the airway.
