# Supplementary material for: Testing the effects of combining azithromycin with inhaled tobramycin for P. aeruginosa in cystic fibrosis; a randomized, controlled clinical trial
Source: Thorax. Author manuscript; Available in PMC 2022 Jun 1. (PMC9043040; doi:10.1136/thoraxjnl-2021-217782)
Supplement: Supp1 [file NIHMS1748397-supplement-Supp1.pdf]

Online Supplement

Testing the effects of combining azithromycin with inhaled tobramycin in people with cystic fibrosis and P. aeruginosa

TEACH: A double-blind, randomized, placebo-controlled, multicenter clinical trial

Registered on Clinicaltrials.gov NCT02677701 including the full study protocol and statistical analysis plan approved by an independent DSMB and the NIH/NHLBI prior to study initiation.

Table E1. Inclusion and Exclusion Criteria

Inclusion Criteria:

- 12 years old or older
- documented diagnosis of cystic fibrosis
- written informed consent (and assent when applicable)
- at least two respiratory cultures growing P. aeruginosa within the last 12 months
- FEV1% predicted between 25-100%
- use of at least two cycles of inhaled tobramycin within the last 24 weeks
- Off TISP and other inhaled anti-pseudomonal antibiotics for at least 2 weeks at Visit 1 and remain off of any inhaled antibiotics for an additional 2 weeks before starting inhaled tobramycin
- most recent liver function test results less than 4 times the upper limit of normal, obtained within the last 12 months
- prior or current use of azithromycin for at least four consecutive weeks
- stable clinical status and therapeutic regimen

Exclusion Criteria:

- weight <40 kg
- positive pregnancy test, lactating, or unwillingness to practice a pre-defined form of contraception, which includes abstinence
- inability to perform reproducible spirometry
- inability or unwillingness to cycle off of inhaled tobramycin for one 4-week period and without use of any additional inhaled antibiotics
- respiratory culture with Burkholderia cepacia complex species within 24 months or with nontuberculous mycobacteria within 18 months of screening
- use of intravenous or oral anti-pseudomonal antibiotics within 4 weeks of screening
- use of investigational therapy within 4 weeks of screening
- use of systemic corticosteroids equivalent to a daily dose more than 10mg of prednisone
- use of nelfinavir, warfarin, haloperidol, or methadone (concern of drug interaction with azithromycin)
- initiation of cystic fibrosis transmembrane conductance regulator (CFTR) modulator therapy within 30 days
- ECG abnormality at screening requiring prompt further medical attention, or QTc interval >480 msec for males and >486 msec for females
- any other condition that, in the opinion of the site investigator, would compromise the safety of the subject or quality of the data

Table E2. Participant baseline characteristics and demographics by treatment group for participants in the microbiology efficacy evaluable population

Characteristic	Azithromycin (N = 29)	Placebo (N = 35)
Age, years	27.9±9.9	27.3±8.3
Age, no. (%)		
≥ 12 to < 18 years	5 (17.2)	5 (14.3)
≥ 18 to < 30 years	14 (48.3)	17 (48.6)
≥ 30 years	10 (34.5)	13 (37.1)
Female, no. (%)	15 (51.7)	15 (42.9)
Race, no. (%)		
Caucasian	24 (82.8)	31 (88.6)
Other*	5 (17.2)	4 (11.4)
Ethnicity, no. (%)		
Hispanic or Latino	6 (20.7)	6 (17.1)
FEV_1, liters	2.38±0.77	2.35±0.85
FEV_1, % predicted †	66.1±18.6	63.3±20.2
FEV_1, % predicted category, n (%)†		
≥ 25% to < 50%	7 (24.1)	10 (28.6)
≥ 50% to < 75%	11 (37.9)	12 (34.3)
≥ 75%	11 (37.9)	13 (37.1)
Height, cm	167.3±9.6	167.6±9.2
Weight, kg	64.6±15.5	63.4±13.9
Genotype, no. (%)		
F508del homozygous	19 (65.5)	22 (62.9)
F508del heterozygous	7 (24.1)	7 (20.0)
Other	3 (10.3)	5 (14.3)
Unavailable	0 (0)	1 (2.9)

Tobramycin Formulation, no. (%)		
Solution	18 (62.1)	20 (57.1)
Powder	11 (37.9)	15 (42.9)
History of Azithromycin Use, no. (%)		
Current Chronic User	25 (86.2)	27 (77.1)
Non-Current Chronic User	4 (13.8)	8 (22.9)
Chronic Medication Use, no. (%)		
Dornase Alfa	25 (86.2)	30 (85.7)
Hypertonic Saline	23 (79.3)	26 (74.3)
High Dose Ibuprofen	1 (3.4)	1 (2.9)
Ivacaftor	1 (3.4)	2 (5.7)
Ivacaftor/Lumacaftor	4 (13.8)	9 (25.7)
Ivacaftor/Tezacaftor	9 (31.0)	13 (37.1)
Elexacaftor/Tezacaftor/Ivacaftor	0 (0)	0 (0)

Plus-minus values are mean ± SD.

*Other includes Black/African American, American Indian/Alaska Native, Asian, Native Hawaiian/Pacific Islander, Unknown, and Other.

† Percent predicted calculated using Global Lung Initiative reference equations.

Table E3. *Pseudomonas aeruginosa* culture results by visit.

	Azithromycin (N = 61)	Placebo (N = 54)	Difference (95% CI)
**Week 0 (Baseline)**			
Number with Results Available	38	39	
Pa Positive Culture, n (%)	31 (81.6%)	31 (79.5%)	2.1% (-15.8%, 19.7%)
**Week 2**			
Number with Results Available	33	37	
Pa Positive Culture, n (%)	27 (81.8%)	35 (94.6%)	-12.8% (-29.4%, 2.8%)
**Change from Week 0 to Week 2**			
Positive at Week 0 and Culture Available at Week 2, n	25	29	
Emergent *Pa* Negative, n (%)	2 (8.0%)	0 (0.0%)	8.0% (-5.0%, 25.0%)
Remained *Pa* Positive, n (%)	23 (92.0%)	29 (100.0%)	-8.0% (-25.0%, 5.0%)
Negative at Week 0 and Culture Available at Week 2, n	6	7	
Emergent *Pa* Positive, n (%)	2 (33.3%)	5 (71.4%)	-38.1% (-69.3%, 13.0%)
Remained *Pa* Negative, n (%)	4 (66.7%)	2 (28.6%)	38.1% (-13.0%, 69.3%)
**Week 6**			
Number with Results Available	36	36	
Pa Positive Culture, n (%)	28 (77.8%)	25 (69.4%)	8.3% (-11.9%, 27.7%)
**Change from Week 0 to Week 6**			
Positive at Week 0 and Culture Available at Week 6, n	22	27	
Emergent *Pa* Negative, n (%)	1 (4.5%)	4 (14.8%)	-10.3% (-28.3%, 9.1%)
Remained *Pa* Positive, n (%)	21 (95.5%)	23 (85.2%)	10.3% (-9.1%, 28.3%)
Negative at Week 0 and Culture Available at Week 6, n	7	8	
Emergent *Pa* Positive, n (%)	1 (14.3%)	1 (12.5%)	1.8% (-34.7%, 40.2%)
Remained *Pa* Negative, n (%)	6 (85.7%)	7 (87.5%)	-1.8% (-40.2%, 34.7%)

Table E4. FEV_1 liters, $ppFEV_1$ and Pa Density changes by chronic use of azithromycin (AZM) at baseline and study visit interval among the primary efficacy m-ITT population and subgroup providing sputum. Data are not adjusted for stratification factors or week 2 values.

**m-ITT Study Population – FEV_1 liters**	**Chronic AZM Users**			**Non-chronic AZM Users**		
Relative (%) change in FEV_1 liters	**AZM**	**Placebo**	**Difference: AZM – Placebo (95% CI)**	**AZM**	**Placebo**	**Difference: AZM – Placebo (95% CI)**
**from Week 0 to Week 6**	46	41	5.61 (1.34, 9.88)	10	11	-4.05 (-14.96, 6.86)
**from Week 0 to Week 2**	46	41	3.16 (-0.26, 6.57)	10	11	-1.04 (-11.21, 9.14)
**from Week 2 to Week 6**	46	41	2.56 (-2.07, 7.18)	10	11	-3.50 (-10.44, 3.45)

**m-ITT Study Population – $ppFEV_1$**	**Chronic AZM Users**			**Non-chronic AZM Users**		
Change in $ppFEV_1$	**AZM**	**Placebo**	**Difference: AZM – Placebo (95% CI)**	**AZM**	**Placebo**	**Difference: AZM – Placebo (95% CI)**
**from Week 0 to Week 6**	46	41	3.83 (0.66, 7.01)	10	11	-2.85 (-8.29, 2.60)
**from Week 0 to Week 2**	46	41	1.28 (-1.02, 3.58)	10	11	0.41 (-5.16, 5.98)
**from Week 2 to Week 6**	46	41	2.55 (-0.23, 5.33)	10	11	-3.26 (-7.73, 1.22)

**Microbiology Efficacy Subgroup – FEV_1 liters**	**Chronic AZM Users**			**Non-chronic AZM Users**		
Relative (%) change in FEV_1 liters	**AZM**	**Placebo**	**Difference: (AZM – Placebo) (95% CI)**	**AZM**	**Placebo**	**Difference: (AZM – Placebo) (95% CI)**
**from Week 0 to Week 6**	25	27	7.18 (1.05, 13.32)	4	8	-2.00 (-28.58, 24.59)
**from Week 0 to Week 2**	25	27	7.50 (2.38, 12.62)	4	8	-3.69 (-25.26, 17.87)
**from Week 2 to Week 6**	25	27	-0.13 (-6.76, 6.50)	4	8	0.78 (-8.29, 9.84)

**Microbiology Efficacy Subgroup – $ppFEV_1$**	**Chronic AZM Users**			**Non-chronic AZM Users**		
Change in $ppFEV_1$	**AZM**	**Placebo**	**Difference: AZM – Placebo (95% CI)**	**AZM**	**Placebo**	**Difference: AZM – Placebo (95% CI)**
**from Week 0 to Week 6**	25	27	5.13 (0.64, 9.63)	4	8	-1.95 (-14.32, 10.41)
**from Week 0 to Week 2**	25	27	3.80 (0.45, 7.14)	4	8	-1.82 (-13.02, 9.38)
**from Week 2 to Week 6**	25	27	1.34 (-2.53, 5.21)	4	8	-0.14 (-4.95, 4.68)

**Microbiology Efficacy Subgroup – *P.a.* Density**	**Chronic AZM Users**			**Non-chronic AZM Users**		
Change in *Pa* density (log_{10}CFUs/mL)	**AZM**	**Placebo**	**Difference: AZM – Placebo (95% CI)**	**AZM**	**Placebo**	**Difference: AZM – Placebo (95% CI)**
**from Week 0 to Week 6**	25	27	1.01 (0.18, 1.85)	4	8	-0.40 (-1.99, 1.19)
**from Week 0 to Week 2**	22	26	0.42 (-0.47, 1.30)	4	8	-0.40 (-1.37, 0.57)
**from Week 2 to Week 6**	22	26	0.71 (-0.11, 1.53)	4	8	0.00 (-1.22, 1.23)

Table E5. Rate of adverse events per participant-weeks of follow-up by system organ class. Rates were calculated as the number of events divided by the total number of follow-up weeks in the respective treatment group.

System Organ Class	Azithromycin (N = 61)		Placebo (N = 54)		
	AEs	Rate	AEs	Rate	Rate Ratio (95% CI)
Ear and labyrinth disorders	0	0	2	0.0058	-
Eye disorders	2	0.0054	4	0.0116	0.47 (0.06, 2.40)
Gastrointestinal disorders	18	0.0487	16	0.0462	1.05 (0.54, 2.09)
General disorders and administration site conditions	9	0.0244	6	0.0173	1.41 (0.51, 4.19)
Infections and infestations	4	0.0108	5	0.0144	0.75 (0.19, 2.83)
Injury, poisoning and procedural complications	1	0.0027	1	0.0029	0.94 (0.04, 23.68)
Investigations	3	0.0081	2	0.0058	1.41 (0.23, 10.67)
Metabolism and nutrition disorders	1	0.0027	2	0.0058	0.47 (0.02, 4.89)
Musculoskeletal and connective tissue disorders	3	0.0081	3	0.0087	0.94 (0.17, 5.06)
Nervous system disorders	17	0.0460	14	0.0404	1.14 (0.56, 2.35)
Psychiatric disorders	1	0.0027	1	0.0029	0.94 (0.04, 23.68)
Renal and urinary disorders	1	0.0027	0	0	-
Reproductive system and breast disorders	2	0.0054	2	0.0058	0.94 (0.11, 7.81)
Respiratory, thoracic and mediastinal disorders	42	0.1137	74	0.2138	0.53 (0.36, 0.77)
Skin and subcutaneous tissue disorders	5	0.0135	3	0.0087	1.56 (0.38, 7.62)
Vascular disorders	0	0	1	0.0029	-

Figure E1. Sensitivity analyses of the primary endpoint regression model of the mean relative (%) change from baseline in FEV_1 liters, AZM=Azithromycin. The “PE + Study Site Adjustment” model added study site as a random effect to the primary endpoint regression model, the “PE + Missing Data Imputation” model used the least favorable arm imputation method to impute missing primary endpoint values with the mean change from the treatment group with the worst change in the observed case analysis, and the “PE + Additional Covariate Adjustment” model added further baseline covariates to the primary endpoint regression model, including age category (≥12-18 years, ≥18-30 years, ≥30 years), Sex (Male, Female), and CFTR modulator use (Yes, No).

Analysis Model	Population	AZM N	Placebo N	6-Week Difference (AZM - Placebo)
Primary Endpoint (PE)	m−ITT	56	52	3.44
PE	Per−protocol	43	40	3.54
PE + Study Site Adjustment	m−ITT	56	52	3.41
PE + Missing Data Imputation	m−ITT	61	54	3.25
PE + Additional Covariate Adjustment	m−ITT	56	52	2.58

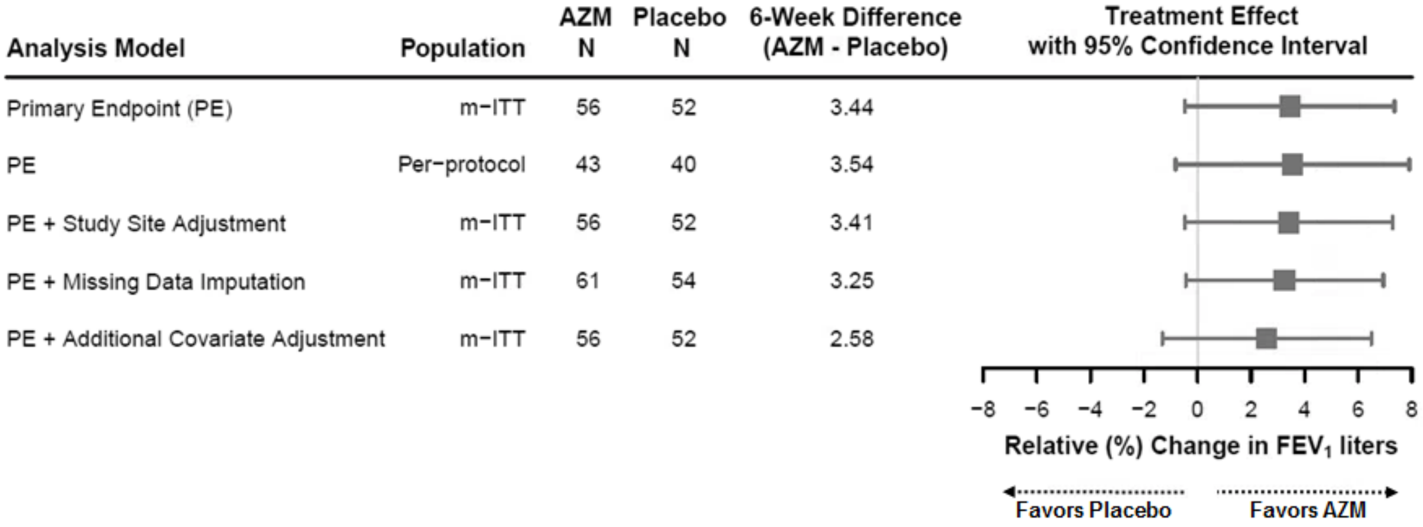

Figure E2. Heterogeneity of relative (%) change in FEV_1 liters from baseline to Week 6 at the participant level, AZM=Azithromycin. Shading refers to self-reported azithromycin (AZM) use at enrollment.

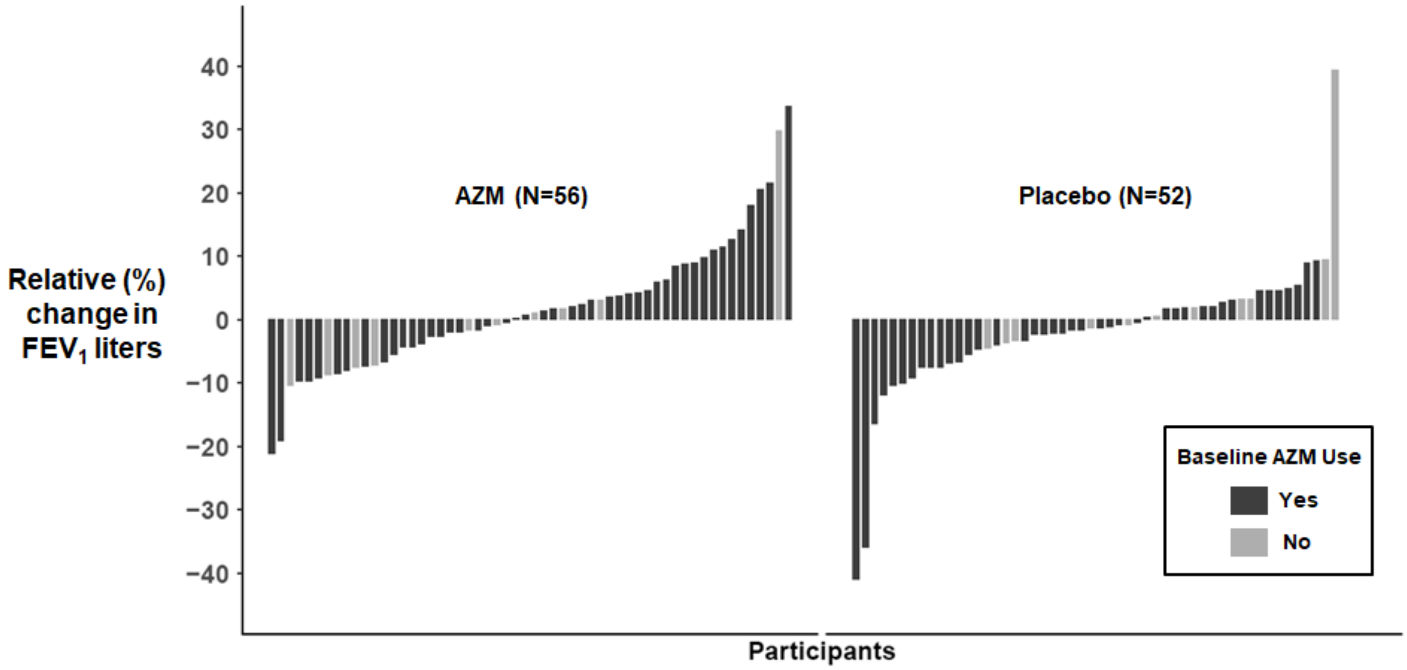

Figure E3. Mean changes in weight (kg) from baseline. Error bars are 95% confidence intervals, AZM=Azithromycin.

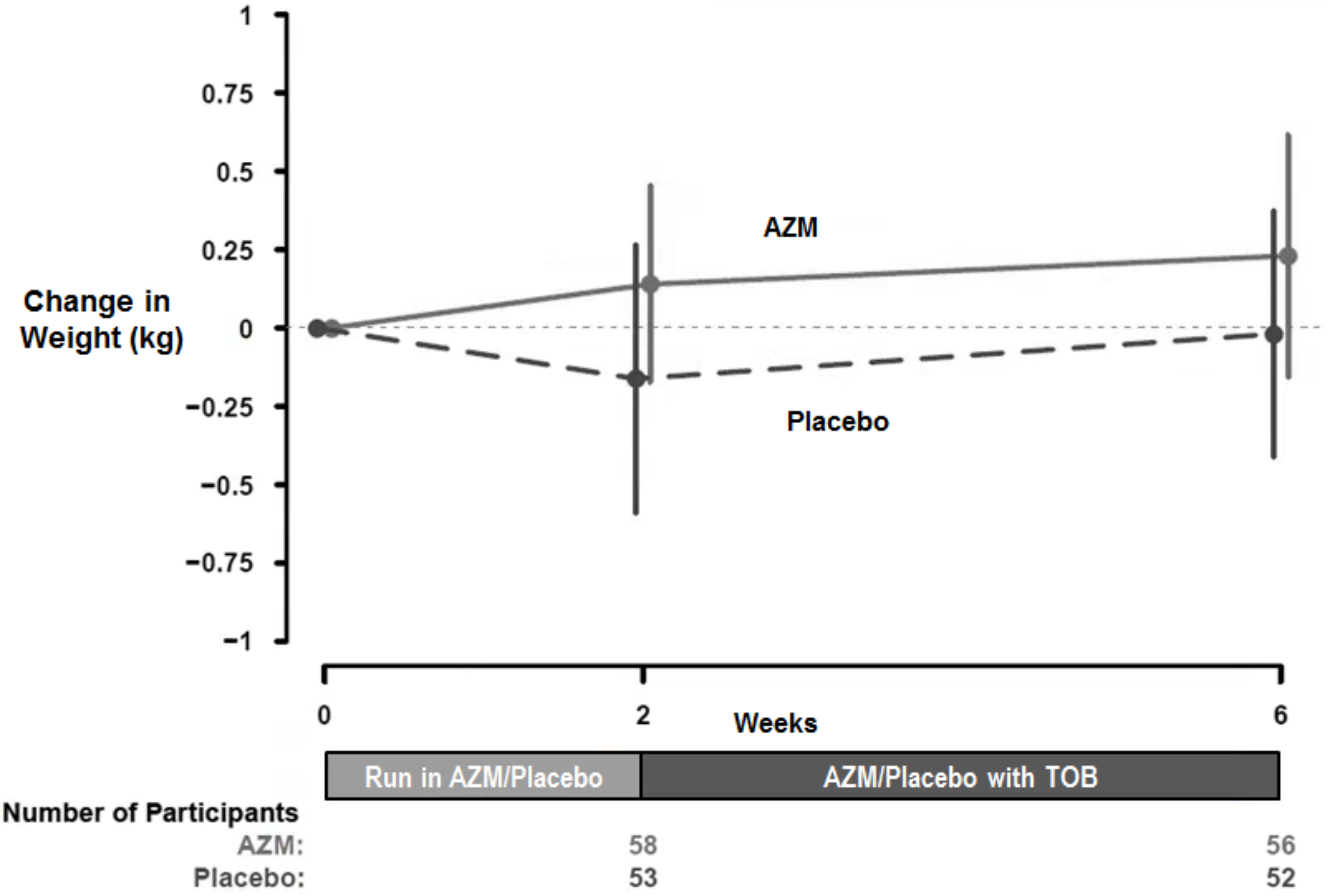

Figure E4. Patient reported respiratory symptom scores: (A) Mean Change in CFQ-R Respiratory Symptom Score (RSS) from baseline and (B) Mean change in CFRSD-Chronic Respiratory Infection Symptom Score (CRISS) from baseline. Error bars are 95% confidence intervals, AZM=Azithromycin.

A

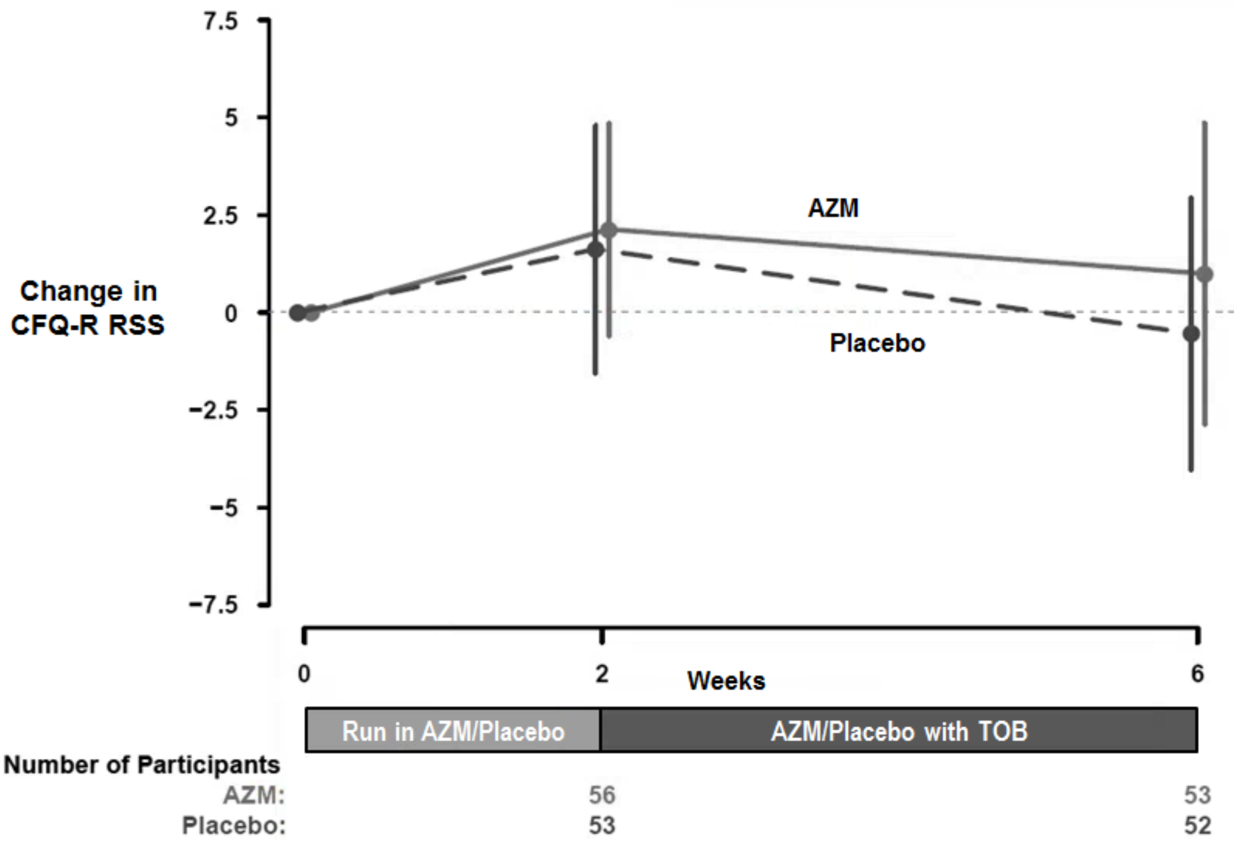

B

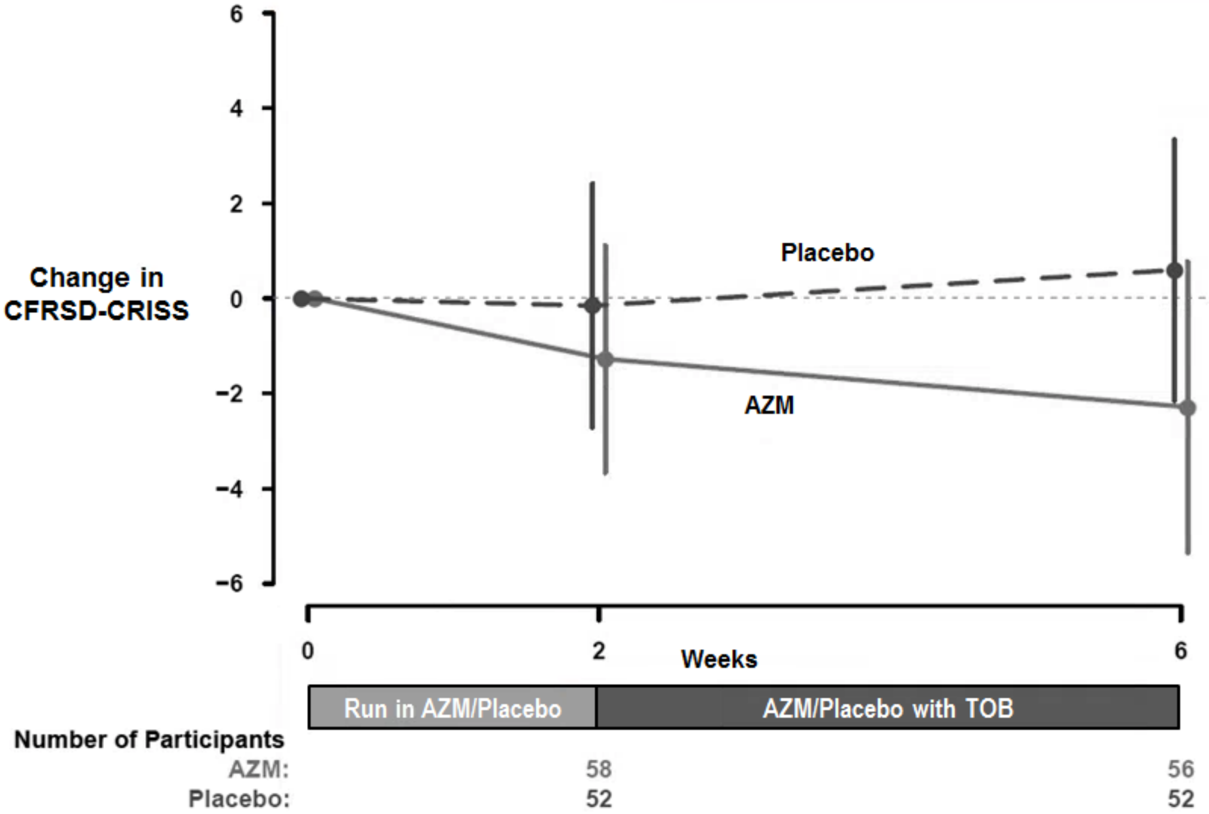

Figure E5. Mean relative (%) change from baseline in FEV_1 liters among the microbiology efficacy population. Error bars are 95% confidence intervals, AZM=Azithromycin.

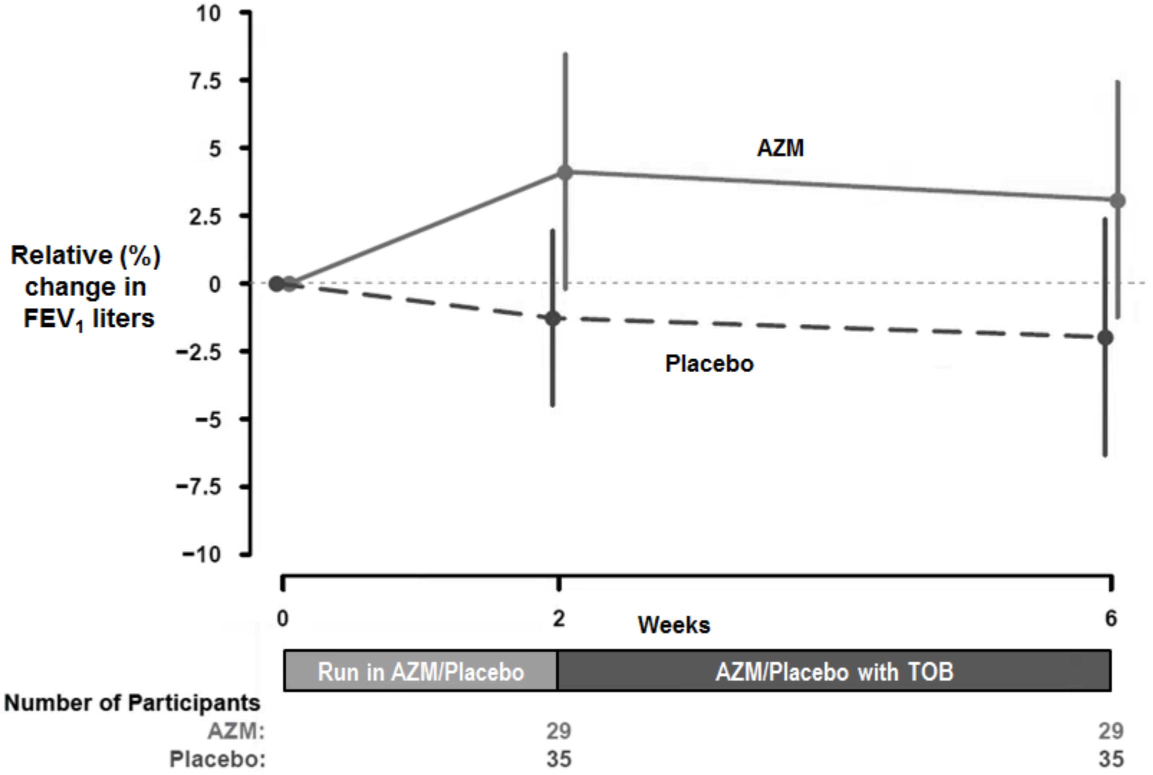

Figure E6. Mean absolute change from baseline in *Pa* density (log_{10}CFUs/mL) among subgroups, AZM=Azithromycin.

Category	Subgroup	AZM N	Placebo N	6-Week Difference (AZM - Placebo)
Overall		29	35	0.79
Age	≥ 12 to < 18 years	5	5	0.56
	≥ 18 to < 30 years	14	17	1.24
	≥ 30 years	10	13	0.24
Sex	Male	14	20	0.44
	Female	15	15	0.98
Baseline FEV_1 % Predicted	≥ 25% to < 50%	7	10	0.41
	≥ 50% to < 75%	11	12	0.64
	≥ 75%	11	13	1.21
Chronic Oral Azithromycin Use	Current User	25	27	1.01
	Non−Current User	4	8	−0.40
Inhaled Tobramycin Formulation	TIS	18	20	1.07
	TIP	11	15	0.31

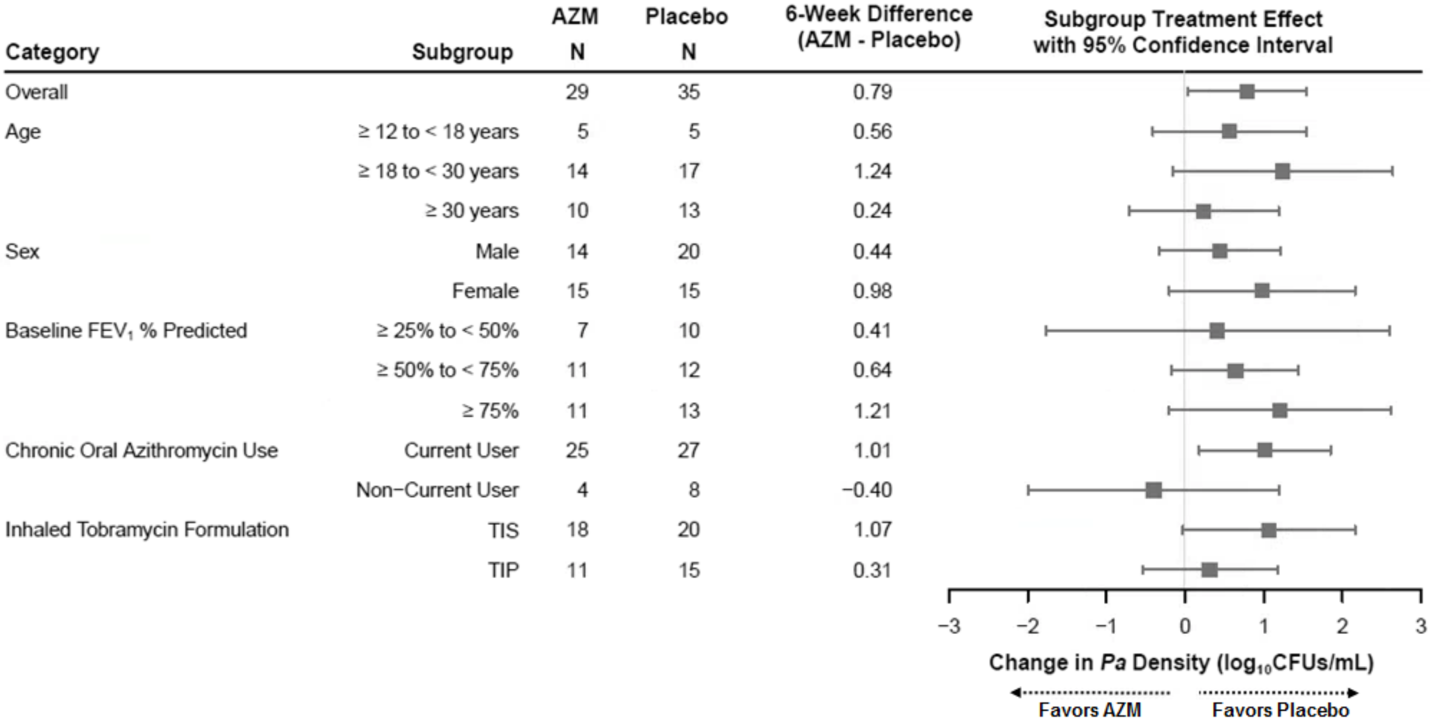

Figure E7. Scatterplot of 6-week relative (%) changes in FEV_1 liters versus 6-week absolute changes in *Pa* density (log_{10}CFUs/mL).

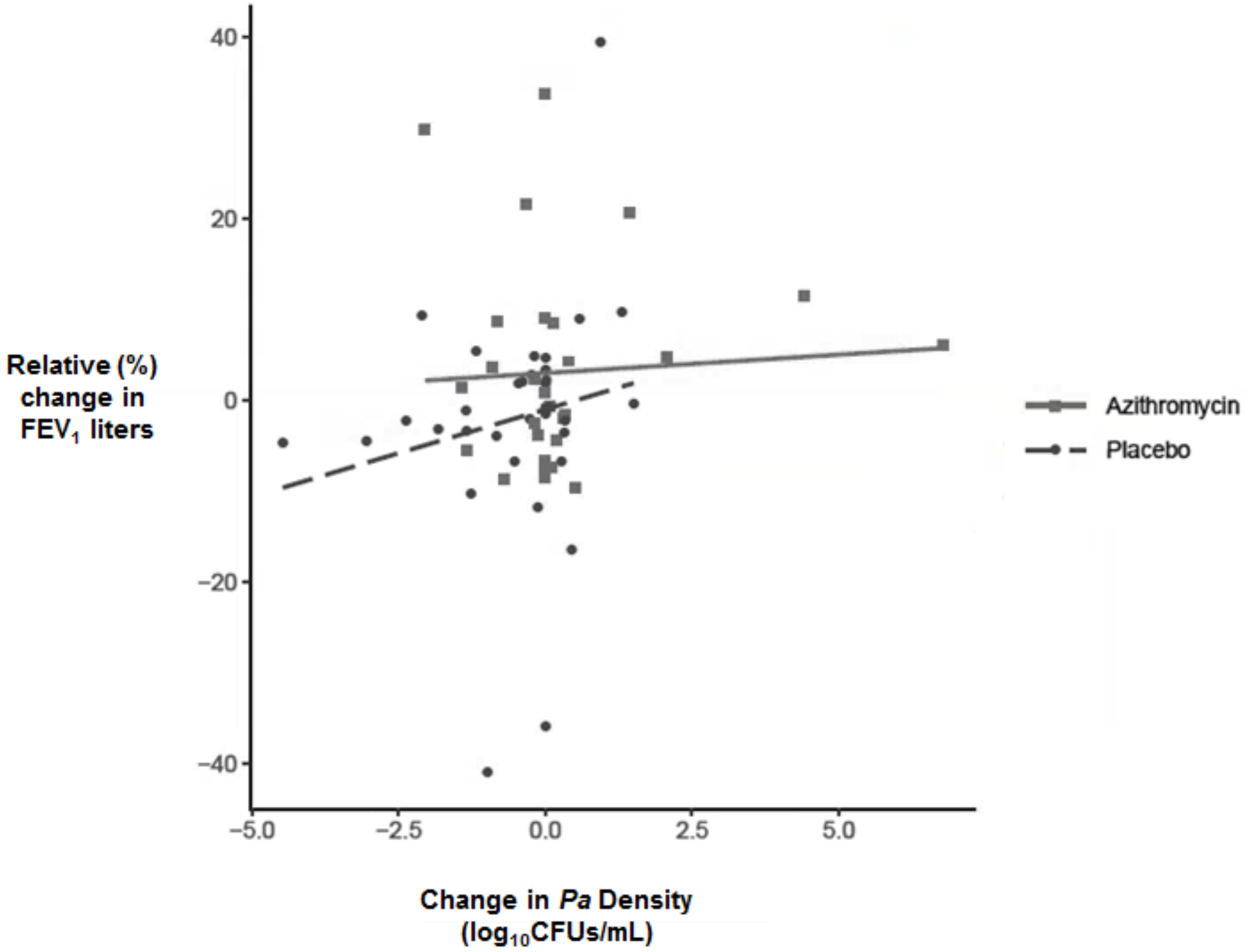

Supplemental Methods

Study Drug

Generic azithromycin tablets (500mg) were obtained and over-encapsulated to matched placebo capsules of very similar weight and appearance (Catalent Pharma Solutions, Somerset, NJ and Univ of Iowa Research Services). The process was coordinated by a central research pharamacist at Seattle Children's Hospital, and study drug was provided in barcoded bottles to site research pharmacies to maintain blinding for other site research staff and investigators. Participants in the 6-week study received only placebo or over-encapsulated azithromycin. Azithromycin drug activity was periodically measured throughout to ensure retained antibiotic effect (National Jewish Laboratory, Denver, CO).

Sputum Collection, Processing, and *P. aeruginosa* assessment:

Sputum was collected by spontaneous expectoration at study sites into sterile collection and mixing tubes. An equal volume of sterile TCEP solution was added to the sputum samples in the tube, and the samples were homogenized using an IKA Ultra Turrax® tube drive system within the same collection tube at 2000 rpm for 10 minutes, reversing direction of mixing every 60 seconds. 1mL of homogenized sputum sample was then pipetted by sterile technique into each of two cryovials containing 0.8mL sterile glycerol and 0.8mL RNAprotect® Bacterial Reagent (Qiagen). Both cryovials were inverted ≥10 times to mix the solution with the homogenized sputum before being placed at -70 to -80C storage. Periodically during the trial, frozen samples were shipped to the Singh Laboratory at University of Washington where samples were thawed in batch and plated by serial dilution on MacConkey Agar to determine *P. aeruginosa* bacterial density. Laboratory personnel and Dr. Singh remained blinded to treatment assignment until the trial was completed and all microbiology data were provided to the coordinating center using study ID numbers.
